# Psychological profile of the bariatric surgery candidates in a spanish hospital in 2020: a descriptive study

**DOI:** 10.1192/j.eurpsy.2021.1734

**Published:** 2021-08-13

**Authors:** N.M. Casado Espada, C. Ortiz-Fune, M. Bersabe-Pérez, S. Delgado-Perales, S. Díaz-Trejo, D. González-Parra, C. Roncero

**Affiliations:** 1 Psychiatry, Psychiatry, Salamanca University Healthcare Complex; Psychiatric Unit. School of Medicine. University of Salamanca (Spain); Institute of Biomedicine of Salamanca (IBSAL), University of Salamanca, Salamanca, Spain., Salamanca, Spain; 2 Psychiatry, University of Salamanca Healthcare Complex, Salamanca, Spain; 3 Psychiatry, University of Salamanca Healthcare Complex. Institute of Biomedicine of Salamanca, Salamanca, Spain

**Keywords:** obesity, Bariatric surgery, PSYCHOLOGICAL PROFILE, psychiatric comorbidity

## Abstract

**Introduction:**

Previous research has found that candidates for bariatric surgery usually present anxiety, depression, personality disorders and/or a tendency to binge eating. The situation related with the pandemic and the lockdowns during the 2020 are possible aggravating factors for these characteristics.

**Objectives:**

To study the more important psychological characteristics presented by candidates for bariatric surgery.

**Methods:**

40 people between 29 and 65 years old (M=46.4, SD=9.1; 37.5% male, 62.5% female) were evaluated between July and December of 2020. The assessment consisted in an interview carried out by a clinical psychologist, and a pool of questionnaires to evaluate depression and anxiety symptoms (Beck Depression Inventory, BDI; and the Goldberg Anxiety and Depression Scale, GADS) the existence of a binge eating pattern (the Binge Eating Scale; BES) and personality traits (the Salamanca Screening Test).

**Results:**

The 25% of the sample had previous mental health antecedents. Eight people disclosed to feel stress in relation with the COVID-19, and 18 presented an emotional regulation strategy using food during the lockdown. 62.5% scored above the cut-off point on the BDI (mild=27.5%, moderate=20%, severe=15%) and a 40% and a 47.5% did it for the anxiety and the depression (respectively) GADS subscales. 20% presented a binge eating pattern according with the BES. Most common personality traits were histrionic (50%), emotionally unstable impulsive type (45%), and anxious (42.5%).

**Conclusions:**

These findings support the previous scientific literature. Psychological intervention programs may be considered to guarantee the surgery’s success, especially when adverse contextual circumstances are presented.

**Disclosure:**

No significant relationships.

